# Canadian Regulatory and Health Technology Assessment for Malignant Hematology and Oncology Indications Compared With the US Food and Drug Administration Accelerated Approval Program

**DOI:** 10.1001/jamanetworkopen.2021.20301

**Published:** 2021-08-11

**Authors:** Cheryl Ho, Howard J. Lim, Dean A. Regier

**Affiliations:** 1 Department of Medical Oncology, BC Cancer, Vancouver, British Columbia, Canada; 2 Department of Medicine, University of British Columbia, Vancouver, British Columbia, Canada; 3 Cancer Control Research, BC Cancer, Vancouver, British Columbia, Canada; 4 School of Population and Public Health, University of British Columbia, Vancouver, British Columbia, Canada

## Abstract

This quality improvement study compares US Food and Drug Administration accelerated approvals with Canadian health and technology assessment approvals and timelines for malignant hematology and oncology treatments.

## Introduction

For serious or life-threatening diseases such as cancer, there is tension between standard regulatory approval processes and patients’ demand for early access to treatments. To address this issue, the US Food and Drug Administration (FDA) initiated the Accelerated Approval (AA) expedited pathway.^1^ AA enables funded drug access through Medicare.

In Canada, a similar expedited Health Canada (HC) pathway exists, but regulatory approval does not guarantee reimbursement. Since 2011, national health technology assessment (HTA) has taken place through the pan-Canadian Oncology Drug Review (pCODR) to facilitate funding decisions.^2^ Drug pricing is negotiated at a national level after a positive pCODR recommendation, followed by formulary listing.

We hypothesize that Canadians have delayed and reduced access to FDA AA drugs. This quality improvement study compares FDA AA with Canadian approvals and timelines for hematology and oncology treatments.

## Methods

This quality improvement study follows the Strengthening the Reporting of Observational Studies in Epidemiology (STROBE) reporting guidelines. This study was exempt from institutional research ethics board review because no patient data were involved, in accordance with 45 CFR §46.

The FDA public electronic database was searched for the combination of AA and malignant hematology or oncology indications from January 2000 to December 2019.^3^ HC databases were searched to identify whether a parallel application was submitted with relevant dates collected. HTAs by pCODR were reviewed from July 2011 (inception of the formalized national process) to December 2019 with relevant dates collected. Outcomes included the median (interquartile range [IQR]) time for each step from AA to Canadian funding and submission or approval rates in Canada. Data analysis was performed in February 2021 using SPSS statistical software version 27 (IBM).

## Results

**Figure 1.**
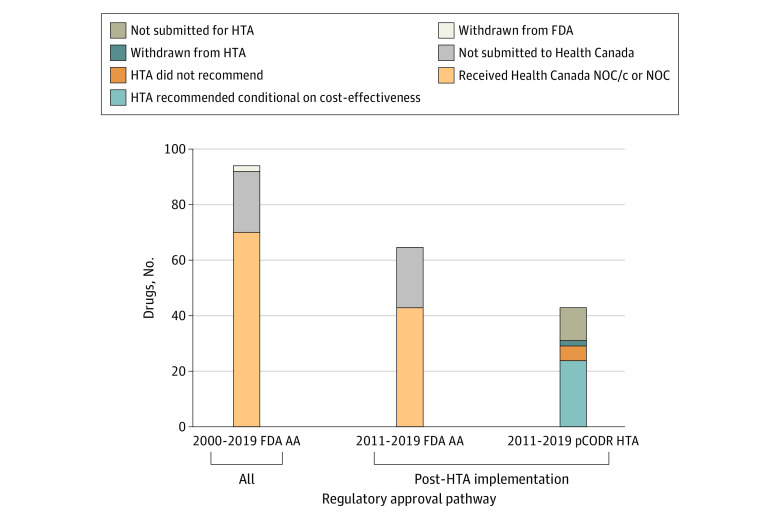
Pan-Canadian Oncology Drug Review (pCODR) Health Technology Assessment (HTA) and US Food and Drug Administration (FDA) Accelerated Approval (AA) Regulatory Pathways for Drugs to Treat Hematological and Oncological Malignant Entities, 2000 to 2019 NOC indicates notice of compliance; NOC/c, notice of compliance with conditions.

**Figure 2.**
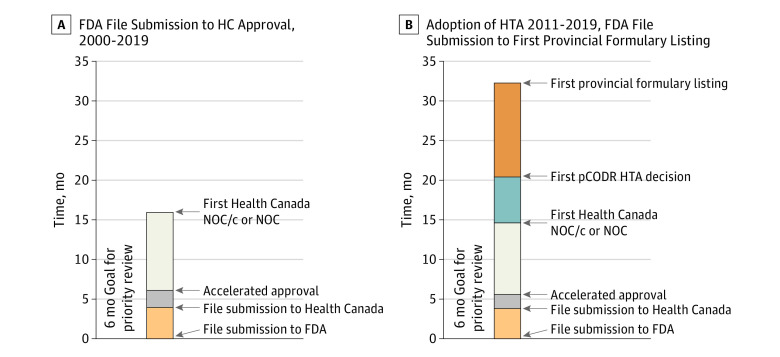
Timeline From US Food and Drug Administration (FDA) File Submission to Health Canada (HC) Approval From 2000 to 2019 and From Adoption of Health Technology Assessment (HTA) From 2011 to 2019 and FDA File Submission to First Provincial Formulary Listing Graphs show median duration of approvals in months. NOC indicates notice of compliance; NOC/c, notice of compliance with conditions; pCODR, Pan-Canadian Oncology Drug Review.

From a regulatory perspective, between January 2000 and December 2019, there were 94 AAs for malignant hematology and oncology indications, 2 of which were subsequently withdrawn. Of these, 70 received regulatory approval in Canada and 22 were not filed (Figure 1). The median (IQR) time from HC submission to AA was 2.1 (2.1-3.4) months, and the median (IQR) time from AA to HC approval was 9.9 (8.6-13.6) months (Figure 2).

From an HTA adoption perspective, from July 2011 to December 2019, there were 65 AA and 43 HC approvals. On final HTA assessment, 24 were recommended conditionally on cost-effectiveness, 5 were not recommended, 2 were withdrawn, and 12 were not submitted. The median (IQR) time from HC approval to first pCODR recommendation was 5.8 (4.5-8.2) months, and the median (IQR) time from pCODR recommendation to first formulary listing was 12.0 (9.8-16.2) months (Figure 2). The median (IQR) time from AA to first formulary listing for HTA-reviewed drugs was 34.0 (24.9-38.5) months.

## Discussion

In this quality improvement study, a review of 20 years of FDA AAs showed that Canadian regulatory approvals aligned with the FDA decisions, but the numbers of submissions to HC and HTA processes were lower in Canada. There was an almost 3-year difference between AA and funded access to treatment for Canadians.

In Canada’s publicly funded health care system, HTA includes systematic evaluation of both clinical effectiveness and cost-effectiveness to optimize health outcomes and allocation of scarce resources. The reason for discordance between AA and Canadian HC and HTA submission is multifactorial and may include the uncertainty around comparative clinical benefits.

Time to access in Canada lags behind that in the US because of regulatory and HTA processes. Project Orbis, whereby multiple regulatory agencies review oncology applications, may reduce delays.^4^ Furthermore, to shorten timelines, pCODR has aligned reviews between HC and HTA.^5^ Multiple procedural changes have been implemented to reducing delays for Canadians.^6^ The limitations of this study include the inability to determine the rationale for pharmaceutical submission decision-making and the factors that may have been associated with delays within regulatory and HTA processes.

Our study highlights challenges that Canadians face in terms of timely access to promising new AA cancer therapies. Nonsimultaneous regulatory submissions followed by HTA result in funded access almost 3 years after AA. Access in Canada, however, is associated with greater certainty of clinical patient benefit, cost-effectiveness, and health care system sustainability. Collaboration among regulators and health care partners will help improve Canadian patient access.
